# How Preferred Brands Relate to the Self: The Effect of Brand Preference, Product Involvement, and Information Valence on Brand-Related Memory

**DOI:** 10.3389/fpsyg.2019.00783

**Published:** 2019-04-26

**Authors:** Rui Feng, Weijun Ma, Ruobing Liu, Miao Zhang, Ziyi Zheng, Ting Qing, Juzhe Xi, Xinzhen Lai, Cen Qian

**Affiliations:** ^1^Department of Marketing, School of Management, Shanghai Business School, Shanghai, China; ^2^Shanghai Changning-ECNU Mental Health Center, School of Psychology and Cognitive Science, East China Normal University, Shanghai, China

**Keywords:** self-reference effect, self-expansion model, brand preference, product involvement, information valence

## Abstract

This study adopted the paradigm of the self-reference effect to explore how brand preference, product involvement, and information valence affects brand-related memory by three experiments. Experiment 1 examined memory differences between positive/negative information of self-/other-preferred brands. Results showed increased memory of positive words (i.e., the effect of information valence) in the self-preferred brand group, yet memory of self-preferred brands was poorer than that of other-preferred brands. Experiment 2 examined effects of degree of brand preference and information valence, and revealed a positive association between degree of preference and memory of brand-related positive words. Experiment 3 explored the effects of brand preference and product involvement. Results showed that the memory of high-preference brands was stronger in the high-involvement group. Additionally, product involvement demonstrated a significant positive correlation with memory. The observed effects of information valence, especially in self-preference (Experiment 1) and high-preference (Experiment 2) conditions, can be explained by self-schema and mnemic neglect theories. The increased memory of highly preferred brands in a high-involvement condition can be explained by intimacy and self-expansion models (Experiment 3).

## Introduction

At the mention of two different cellphone brands, such as Apple and Samsung, two different adjectives are likely to come to mind that describe the brands. The reason why different memories of various brands form, and exactly how these memories differ, remains to be elucidated. The positive or negative memories consumers hold regarding various products and brands strongly contribute their final purchase decisions. As self-schemas effectively influence memory, the choice making process of consumers is actually a process of seeking psychological identity and self-expression. This is similar to a concept presented by Aaker and Fournier (1995), stating that when people build a relationship with a brand, they actually view the brand as a character, a partner, and a person, and that they choose to build relationships with brands because such relationships help them develop a sense of who they are (Aaker and Fournier, 1995; Fournier, 1998).

The “self” is a remarkably important topic in social psychology. This is especially true of the self-reference effect (SRE; Rogers et al., 1977). While this effect has been widely applied in studies of interpersonal relationships, it has not been applied to research examining self-brand relationships. The current manuscript uses the Remember/Knowledge (R/K) paradigm of the SRE to determine how products and brands influence memory, exploiting application domains for the SRE and providing marketing strategy insight.

## Self-Reference Effect (SRE)

The SRE (Rogers et al., 1977) refers to the phenomenon of better memory being observed in self-referential tasks than other tasks. The purported mechanism responsible for this effect is the more elaborate and organizational processing of self-related information when compared to other information. Previous research of this effect has followed two different paradigms, specifically, the classical SRE paradigm, and the R/K paradigm (e.g., Liu and Zhu, 2002). In the classical SRE paradigm, subjects rate adjectives on four tasks designed to force varying kinds of encoding: structural, phonemic, semantic, and self-referential, and then are asked to freely recall the rated words (e.g., Rogers et al., 1977). The R/K paradigm is more sensitive than the classical (e.g., Conway and Dewhurst, 1995), asking subjects to first identify old items in a recognition test, and then indicate which items are accompanied by a recollective experience (“Remember” responses) and which are recognized on some other basis (“Know” responses). The SRE is primarily shown in “R” responses, as previous research (Zhang et al., 2005) has verified that “R” responses reflect the SRE more effectively and more sensitively than “K” responses.

### The Effect of Intimacy on SRE

A number of studies have consistently demonstrated that intimacy has an important effect on SRE. Specifically, referring to highly intimate others promotes a memory effect that is almost as strong as self-reference (Symons and Johnson, 1997; Zhou and Su, 2008; Xiong et al., 2012). This can be reasonably explained by the self-expansion model proposed by Aron and Aron (1986). The model suggests that human beings have a motivation to include other people, including their resources, concept, and identity, into the self-concept in an effort to increase their own self-efficacy (Aron and Aron, 1986; Aron et al., 1991; Jia and Shi, 2012), and that, to the degree that a relationship is intimate, it is likely to overlap with one’s self-representation. In other words, the higher the degree of intimacy, the higher the degree of self-inclusion, which leads to better memory effect.

### The Effect of Information Valence on SRE

Rogers et al. (1977) pointed out that people had better memory of self-related trait adjectives than other words. Kuiper and Derry’s (1982) further study found that, in self-referential conditions, people’s memory of positive adjectives was enhanced when compared to negative adjectives. A study by Argembeau et al. (2005) also supported this notion, in that positive trait information was typically better recalled than negative trait information when encoded in reference to the self, but not when encoded in reference to someone else or when processed for general meaning. Green and Sedikides (2004) discovered that information that threatened positive aspects of the self (e.g., “I would be unfaithful in an intimate relationship”) was less likely to be remembered in self-referential conditions, but not under other conditions.

Self-schema theory can explain the effect of enhanced memory of positive self-referential adjectives. The self-schema theory raised by Markus (1977) points out that self-schemata are cognitive generalizations about the self, derived from past experience, that organize and guide the processing of the self-related information contained in an individual’s social experience. For example, one study found that self-schemata facilitated the processing of information about the self, contained easily retrievable behavioral evidence, and provided a basis for the confident self-prediction of behavior on schema-related dimensions (Markus and Wurf, 1987). This also explains the general mechanism of SRE to some extent, because individuals are more sensitive to self-related information. Liao and Chen (2006) found that self-schemata influenced mental health. While patients with depression had underlying negative self-schemata, psychologically healthy people demonstrated rather positive self-schemata, such as positive self-concept and high self-esteem. Self-esteem was also found to influence memory, as people with high self-esteem tend to show greater preference for self-related positive information (He and Qin, 2009). Since self-related positive information results in elaborate processing from one’s self-schemata, the schemata activate an information network to more effectively extract the memory in recognition phases, leading to enhanced memory.

In addition, the mnemic neglect model (Sedikides and Green, 2000; Sedikides and Green, 2004) can also explain differences in memory of positive and negative words in the SRE. The model postulates a two-stage sequence of processing self-referential feedback. In Stage 1, the individual appraises the plausibility of enacting the behavior based upon general self-knowledge. That is, the individual compares the behavior to semantic information about the self. Since the self is usually a positive, complicated, and motivated structure (Gaertner et al., 2002; Sedikides et al., 2003), if the feedback is threatening to the self, processing is largely confined to Stage 1 and does not proceed to Stage 2. In Stage 2, the behavior is further compared to relevant and specific self-knowledge. This leads to negative information “neglected” at Stage 1. From the point of view of self-protection, the model is essentially selective memory that ignores negative self-related information and pays more attention to the positive information (Green and Sedikides, 2004, 2006; Chen and Zhao, 2009). Therefore, according to the model, subjects find it difficult to recall negative words because they have been exposed to less processing.

## Brand Preference and Product Involvement

While many empirical studies have been performed in the field of interpersonal memory, very little research has been conducted examining brand memory. It is possible that SRE paradigms can also be applied to the study of brand-related memory, in which brand preference and product involvement can play the same role intimacy plays in the SRE.

Crites et al. (1994) defined preference as revealed attitudes and tendency toward certain objects. Brand preference can then be understood as the positive evaluation, degree of preference, and purchase predisposition of a specific brand (Zhang and Lei, 2011). This can be influenced by the self, as the self ultimately shapes attitudes. For example, Ji (2008) found that the higher the degree of matching between a brand personality and the consumer personality, the higher the degree of involvement and the higher the degree of brand preference. Although such studies have combined conceptualizations of the self with brand preference, they have remained narrow in scope, focusing solely on the domain of the impact of the self on brand preference, but lacking more profound and extensive exploration.

The previously discussed concept of involvement can be defined as the perceived relevance of an object based on inherent needs, values, and interests (Zaichkowsky, 1985). Any type of product-related information can cause involvement between consumers and products, such as advertisements or product packaging. Consumption behaviors are intricately interwoven with involvement, and, once consumers have formed a stable involvement relationship with a product, they will perform both purchasing and impulsive consumption behaviors.

It is regrettable that the effects of brand preference and product involvement on the brand-related memory remain unexplored. Hence, the current study aimed to fill the research gap by adapting the R/K SRE paradigm to study brand-related memory.

## Research Purposes and Hypotheses

### Research Purposes

In modern life of consumption, the self and brands have a close connection (Angle and Forehand, 2016). The SRE has been applied to many interpersonal relationship studies, such as research of the mother-reference effect (Symons and Johnson, 1997; Zhou and Su, 2008; Xiong et al., 2012), but it rarely appears in research regarding memories of brand information. Zhou et al. (2012) his found that free recall scores of self-owned objects were significantly higher than other-owned objects, showing the ownership effect (i.e., a virtual ownership reaction that occurs merely by use of pronouns such as “my” and “his”), impacted memory. In the present research, the self was combined with brand preference and product involvement to explore the relationships among these factors.

The overall goal of the current research was to provide evidence that brand preference, degree of brand preference, and product involvement would each demonstrate an impact on the memory of relevant product information by using the R/K paradigm of the SRE. Additionally, the current series of studies attempted to test differences in memory of brand-related positive and negative information under the influence of brand preference and product involvement.

### Research Hypotheses

#### Memory of Brand-Related Information

According to the self-expansion model (Aron and Aron, 1986; Aron et al., 1991), the self has a tendency to include preferred brands and other high-involvement products into the self-concept. Analogous to the SRE, individuals should demonstrate a memory advantage for information of preferred brands and high-involvement products. Further, memory of positive and negative words associated with these, and other, products should differ.

##### The effect of brand preference on memory

This research predicted an impact of brand preference on memory of related information. According to Aron’s self-expansion model, people include other people into the self (Aron and Aron, 1986; Aron et al., 1991; Jia and Shi, 2012). It has been suggested that brands are also likely to be included within this framework (Reimann and Aron, 2009). The more self-expansion people feel toward a brand, the more loyal they will be to it. As a result, based on the self-expansion model, highly preferred brands will receive more self-expansion, thus leading to the better memory of relevant information. This effect would be similar to that observed in intimacy.

##### The effect of product involvement on memory

Ji (2008) found that when consumers perceived that a product helped to achieve their ideal image, involvement with the product would increase. Similarly, Chen (2007) also pointed out that if the connection between people and a product became stronger, involvement levels would also rise, leading to various behaviors, such as caring about the product and searching for relevant information. This then results in a stronger emotional investment with the product. With high-involvement products, consumers spend more time comparing among different markets, and when it came to low-involvement products, the time was saved (Clarke and Belk, 1979). High-involvement products are important to consumers and require a large amount of information processing, while low-involvement products are not very important, thus requiring less information processing. Therefore, the elaborately processed information of the former will be more easily extracted during recognition phase than the information processed for the latter.

##### The interaction between brand preference and product involvement

Since intimacy, according to the self-expansion model, has a strong effect on the SRE in relationships among different people, it is possible that brand preference and product involvement will have a similar effect on memory in the relationship between people and objects. Based upon the self-expansion model (Aron and Aron, 1986), high-involvement products will be more deeply included into the concept of the self, leading to more elaborate processing of relevant information. This then suggests that brand preference will have a significant influence on memory under this condition, with the memory of high-preference brands being better than the one of low-preference brands.

Similarly, low-involvement products will be more weakly included into the self-concept. Because of this, relevant information will receive less processing, weakening the influence of brand preference on memory under this condition, presenting as no memory difference between the information of high- and low-preference brands.

##### The effect of information valence on memory

Being analogous to the difference in memory effects between positive and negative words in the SRE (Rogers et al., 1977), the current study suggests that individual’s memory of positive and negative words of products with different levels of product involvement and brand preference will be different, and that this effect can be explained by self-schema theory and the mnemic neglect model. In high-preference brands, individuals will form positive schemata and selectively ignore negative information, thus, in this condition, the memory of positive information for high-preference brands should be significantly better than the memory of negative information. In low-preference brands, memory of positive and negative information should demonstrate no difference. Furthermore, this result should be more noticeable in high-involvement products, as the relevant information will likely receive more complex processing.

### Overview of the Current Experiments

To test the impact of brand preference, product-involvement, and information valence on memory, three experiments were performed. Experiment 1 used the R/K paradigm of SRE as the method and shampoo as the material to compare the memory effects of self-preferred and other-preferred brands. Additionally, the impact of information valence was also measured. It was predicted that the memory of self-preferred brands would be enhanced and that positive information of the brand would demonstrate enhanced memory when compared to negative information.

Experiment 2 also used the shampoo as material, exploring the impact of information valence and the different impacts of high, medium, and low brand preference on memory. It was predicted that a higher degree of brand preference would lead to better memory, and that memory differences between relevant positive and negative information would be even more obvious.

Experiment 3 added the variable of involvement to explore the impact of involvement and brand preference on memory. Specifically, this experiment used shower gels and computers as materials of low and high product involvement, respectively. It was predicted that information regarding the preferred brand with higher product involvement would demonstrate the best level of memory, with a greater difference between positive and negative words.

## Experiment 1: The Effect of Self- and Other-Preferred Brands and Valence of Relevant Information on Memory

This experiment intended to examine whether memory of a self-preferred brand would be enhanced when compared to the memory of an other-preferred brand. Additionally, this experiment explored whether the valence of words (i.e., positive or negative) would show differing effects depending on the preference category.

### Experimental Hypotheses

Individuals have a tendency to incorporate preferred brands into their self-concepts (Aron and Aron, 1986). Therefore, considering this information and the SRE, it was hypothesized that individuals would demonstrate a memory advantage for information of these preferred brands (i.e., brands incorporated into the self) through elaborate and organizational processing.

It was also hypothesized that the memory of positive and negative information of these preferred brands would differ. According to self-schema theory (Markus, 1977), one of the most common self-schemata is maintaining positive self-image. This schema activates a “net” to extract positive information when forming memories. In addition, the mnemic neglect model (Sedikides and Green, 2000; Sedikides and Green, 2004) indicates that people will ignore self-related negative messages. As a result, the memory of positive trait adjectives is better than the memory of negative adjectives when referencing the self, but not when referencing others. Therefore, people will likely also hold a positive schema for preferred brands and ignore their negative aspects, leading to enhanced memory of positive words describing self-preferred brands.

In summary, the hypotheses of Experiment 1 were:

*Hypothesis 1a: The overall recognition and R rates of self-preferred brand information will be significantly higher than that of the other-preferred brand;*
*Hypothesis 1b: Subjects’ brand preference will demonstrate a significant positive correlation with the memory of the product’s relevant information;*
*Hypothesis 1c: The recognition and R rates of positive information will be significantly higher than negative information in the group of self-preferred brands, and no difference will appear in the group of other-preferred brands.*

### Methods

#### Participants

A total of 33 participants (17 males, *M*_age_ = 21.9 years, *SD*_age_ = 1.24) with normal or corrected to normal vision were recruited from universities in Shanghai.

#### Experimental Design

The experiment was a 2 (referential task: self-preferred brand vs. other-preferred brand) × 2 (information valence: positive vs. negative) within-subjects design. Dependent variables were recognition and R/K rates.

#### Materials

The materials of Experiment 1 consist of questionnaire and adjectives. Please see Appendix A for details.

##### Questionnaire

Shampoos are commonly used objects in daily life, which are familiar to people and exhibit a high degree of market concentration (Gu, 2008). Therefore, a questionnaire targeting shampoo brands (e.g., Pantene, Rejoice) was designed to collect data. The questionnaire contained two parts. The first was to confirm and evaluate a self-preferred brand. In this section, subjects were asked to choose their favorite shampoo brand and evaluate it on two dimensions (preference and similarity), with each item ranked on a seven-point, Likert-type scale (1 = *strongly disagree*, 7 = *strongly agree*). The second part was to determine and evaluate an other-preferred brand on the same two dimensions. Specifically, after finishing the first part, subjects were told that their preferred brand was different from the former participant’s choice, and were asked to guess the former’s favorite brand and evaluate it on the same two dimensions.

##### Adjectives

All adjectives used in the paradigm to describe brands were chosen from the Research group of Modern Chinese Glossary of Common Words (2008), half of which were positive and the other half were negative. Words were divided into four groups, balanced in terms of the valence, frequency, and phonetic sequence. Each group had 28 words (14 positive and 14 negative), totaling 112 words in all four groups. Two groups of words were randomly chosen to describe the self- and other-preferred brands in the learning phase, and the other two groups of words were defined as new words in the recognition phase. Word order of appearance was randomly generated by E-prime (E-prime is a psychological experimental operation platform which is an advanced graphic design environment and a cross-platform system to realize computerized behavior research).

#### Experimental Procedure

After finishing the questionnaire, subjects completed the R/K paradigm experiment. The experiment was conducted in three phases: learning phase, interference phase, and testing phase. The first and third phases were completed on E-prime 2.0. The instructions of learning phase and testing phase are showed in Appendix A. The entire experimental protocol lasted for approximately 30 min.

##### Learning phase

Subjects were told that this was an adjective evaluation experiment, and they were to complete two kinds of judging tasks by answering the questions “Is the word xxx (e.g., ‘popular’) appropriate to describe the xxx (self/other-preferred brand chosen by participant)?” Each task involved 28 adjectives (14 positive and 14 negative), for a total of 56 adjectives. According to each participant’s own choices of self- and other-preferred brands in the questionnaire, brand names were modified in E-prime by the experimenter and appeared in the experimental instructions. The appearances of these two tasks were balanced. At the beginning of each task, there were detailed instructions, telling that after the presentation of a fixation point and a blank screen lasting for 500 ms, each adjective would be presented for 2 s. Participants’ reactions were not recorded, as the purpose of this phase was to allow them to learn the words.

##### Interference phase

At the end of the learning phase, subjects were given a 3-min break. Then they were asked to perform 64 mathematical calculations to avoid repetition of the words that appeared in the learning phase. This phase lasted for 7–9 min.

##### Testing phase

After the interference phase, subjects were asked to complete recognition tasks. They were randomly presented 56 old and 56 new words (half positive and half negative). First, subjects were asked to determine if words were “new” or “old.” When words were deemed “old,” participants were asked to judge whether they exactly “Remembered” the words or just “Knew” the words. There was no fixed presentation time for each word, as words were switched when participants pressed a button. Before each word, a fixation point and blank screen was presented for 500 ms.

#### Measures

Dependent variables (i.e., memory results) were the “Recognition rate” (the number of words correctly judged as “old”)/56 (the total number of words in the learning phase), the “R rate” (number of “Remember” words)/56, and the “K rate” (number of words subjects “Know”)/56.

### Results

This study used SPSS 23.0 to analyze data in the questionnaire, recognition rates, and R/K rates.

#### Manipulation Checks

A paired-sample *t*-test was performed to test the preference and familiarity of self-/other-preferred brands. Results indicated a significant difference in degrees of preference [*t*(32) = 5.82, *p* < 0.001, 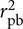 = 0.51] (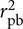 is the effect size reflecting the percentage of variation in the dependent variable which can be explained by the independent variable in *t*-test). The preference of the self-preferred brand (*M* = 6.12, *SD* = 0.78) was much higher than the other-preferred brand (*M* = 4.85, *SD* = 1.13). The familiarity of the self-preferred brand (*M* = 5.12, *SD* = 1.14) was also higher than the other-preferred brand (*M* = 4.73, *SD* = 1.33), but not significantly [*t*(32) = 1.51, *p* > 0.05, 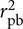 = 0.07]. Therefore, the self-preferred brand and the other-preferred brand were well distinguished on degree of preference.

Moreover, recognition rates of new words were calculated by using the number of the new words that were misjudged as “old” by participants. A within-subject one-way analysis of variance (ANOVA) performed on the recognition rates of new words and old words revealed a significant effect [*F*(1,35) = 433.22, *p* < 0.01]. Recognition rate of old words (*M* = 0.60, *SD* = 0.15) was higher than new words (*M* = 0.24, *SD* = 0.11), indicating an effective manipulation of old and new words.

#### Recognition Rate

Descriptive statistics of recognition rates and R/K rates of different referential task and different information valence are presented in Table 1. A 2 (referential task: self-preferred brand vs. other-preferred brand) × 2 (information valence: positive vs. negative) repeated measures ANOVA on recognition rates revealed a significant main effect of referential task [*F*(1,32) = 10.90, *p* = 0.002, η^2^ = 0.25]. The recognition rate of self-preferred brands (*M* = 0.56, *SD* = 0.17) was lower than that of other-preferred brands (*M* = 0.63, *SD* = 0.15), a result in direct opposition of hypothesis 1a. The main effect of information valence was not statistically significant [*F*(1,32) = 0.73, *p* = 0.40, η^2^ = 0.02].

**Table 1:** The M (SD) of the recognition rate and the R/K rate of different referential tasks and information valence.

Memory indexes	Referential tasks					
	Self-preferred brands			Other-preferred brands		
	Positive words	Negative words	Mean	Positive words	Negative words	Mean
Recognition rate	0.60 (0.18)_1	0.53 (0.23)_2	0.56 (0.17)_3	0.62 (0.17)	0.63 (0.20)	0.63 (0.15)_4
R rate	0.32 (0.19)_1	0.24 (0.21)_2	0.28 (0.18)_3	0.35 (0.20)	0.35 (0.22)	0.35 (0.19)_4
K rate	0.29 (0.16)	0.29 (0.21)	0.29 (0.16)	0.27 (0.17)	0.29 (0.20)	0.28 (0.16)

The interaction between referential task and information valence was significant [*F*(1,32) = 4.46, *p* = 0.04, η^2^ = 0.12]. In the self-referential condition, there was a marginally significant difference between the memory of positive words and negative words [*F*(1,32) = 2.98, *p* = 0.09, η^2^ = 0.09], indicating that the memory of the former was enhanced. In other-referential condition, there was no statistically significant memory difference between two kinds of words [*F*(1,32) = 0.13, *p* = 0.72, η^2^ = 0.004].

#### R Rate

A 2 (referential task: self-preferred brand vs. other-preferred brand) × 2 (information valence: positive vs. negative) repeated measures ANOVA on R rates also revealed a significant main effect of referential task [*F*(1,32) = 14.13, *p* = 0.001, η^2^ = 0.31]. The R rate of self-preferred brands was lower than the one of other-preferred brands. The main effect of information valence in this analysis was also non-significant [*F*(1,32) = 2.23, *p* = 0.15, η^2^ = 0.07].

The interaction between referential task and information valence was significant [*F*(1,32) = 5.33, *p* = 0.03, η^2^ = 0.14]. For a further simple effect analysis, we fixed the referential task and analyzed information valence. In the self-referential condition, memory of positive words was significantly better than the memory of negative words [*F*(1,32) = 5.85, *p* = 0.02, η^2^ = 0.16]. In the other-referential condition, there was no significant memory difference between the two kinds of words [*F*(1,32) = 0.04, *p* = 0.84 η^2^ = 0.001].

#### K Rate

A 2 (referential task: self-preferred brand vs. other-preferred brand) × 2 (information valence: positive vs. negative) repeated measures ANOVA on K rates found no significant main effects of referential task or information valence [*F*_referentialtask_ (1,32) = 0.54, *p*_referentialtask_ = 0.47, η^2^_referentialtask_ = 0.02; *F*_informationvalence_ (1,32) = 0.31, *p*_informationvalence_ = 0.58, η^2^_informationvalence_ = 0.01]. The interaction between referential task and information valence was also non-significant [*F*(1,32) = 0.09, *p* = 0.77, η^2^ = 0.003].

#### Correlations Between Brand Preference and Memory

Correlations among the degree of brand preference, recognition rate, R rate, and K rate are reported in Table 2. The degree of brand preference had a marginally significant negative correlation with the overall recognition rate (*r* = -0.21, *p* < 0.10) and a significant negative correlation with the R rate of negative words (*r* = -0.26, *p* < 0.05), indicating that the higher the brand preference, the lower the overall recognition rate, and the R rate of negative words decreased along with an increase of brand preference. Additionally, the memory of positive words was not significantly related to brand preference.

**Table 2:** Correlations (*r*) among preference, recognition rate, and the R/K rate of positive and negative words.

	Recognition	R rate	K rate	Recognition	Recognition	R rate	R rate	K rate	K rate
	rate of all	of all	of all	rate of PW	rate of NW	of PW	of NW	of PW	of NW
Preference	-0.09	-0.21^†^	0.16	-0.06	-0.08	-0.11	-0.26^∗^	0.08	0.19

*PW, positive words; NW, negative words. ^∗∗∗^*p* < 0.001, ^∗∗^*p* < 0.01, ^∗^*p* < 0.05, ^†^*p* < 0.10*.

### Discussion

The results of Experiment 1 showed that memory effects of shampoo brand information were different under two different referential conditions, an effect in which information valence plays a role. ANOVA revealed that the memory of self-preferred brands was worse than that of other-preferred brands on both overall recognition and R rates. Correlation analysis showed that brand preference demonstrated a negative correlation with R rate, which was consistent with the ANOVA results, and failed to support hypotheses 1a and 1b. Overall, in recognition and R rates, the memory of positive words was better than negative words in the self-referential condition and not in other-referential condition, supporting hypothesis 1c.

Hypotheses 1a and 1b were not supported; the overall recognition and R rates of the information of the self-preferred brand was worse than that of the other-preferred brand. This may be because the familiarity of self-preferred brands was not significantly higher than that of the other-preferred brands, despite the fact that the former demonstrated higher preference than the latter. Liu (2015) found that similarity of materials had a large impact on working memory spans. Therefore, similarity might influence memory levels of different referential tasks (Symons and Johnson, 1997). Correlation analysis also showed that preference was negatively correlated with the R rate of negative information, which meant that the higher the brand preference, the worse the negative information was remembered, and the memory of negative information was so poor that it made the preference negatively correlated with all of the information of the brand. In other words, it was not the self-preferred brand, but rather the negative information of the brand that resulted in poorer memory. Nevertheless, there was no memory difference observed in the K rate, providing evidence in support of assertions made by previous researchers that there is no SRE in K responses.

Experiment 1 also found that, in the self-referential condition, the memory of positive information was enhanced when compared to that of negative information. However, in the other-referential condition, the difference disappeared. This result provides support for hypothesis 1c. Previous studies have suggested that it is difficult for people to remember negative words which may threat their positive self-image, and easy for them to remember positive words which may improve their self-image (Argembeau et al., 2005). The observed difference was not observed in K rate, once again supporting the notion that K rate is not sensitive enough a measure of the SRE.

Since different memory effects of positive and negative information of self-preferred brands were found in Experiment 1, Experiment 2 would remove the other-referential group and divide the referential conditions into the groups of high, moderate, and low levels of self-preference, examining the main effect of the degree of brand preference on memory, and its interaction with information valence.

## Experiment 2: The Effect of the Degree of Brand Preference and Valence of Relevant Information on Memory

This study intended to discuss the impact of the degree of preference and information valence on the memory of brand-related information.

### Experimental Hypotheses

It has been shown that individuals tend to have a better memory of highly intimate others, because of these individuals being more deeply included into the self-concept (Symons and Johnson, 1997; Zhou and Su, 2008; Xiong et al., 2012). Further, individuals have also been found to have better memory of self-related positive words (Kuiper and Derry, 1982). Self-schema theory indicates that self-related positive information can be processed at a higher level. Therefore, the current study predicted that brands are deeply included into the self-concept when a high degree of preference is reported, and thus, would demonstrate better memory effects. Moreover, people’s memory of the brand’s positive information would also be better, and the difference between the memory of positive and negative information would be larger.

To sum up, the hypotheses of Experiment 2 were:

*Hypothesis 2a: The higher the level of preference for a brand that subjects hold, the better overall recognition and R rates of the brand’s relevant information, when compared to lower levels of preference;*
*Hypothesis 2b: Subjects’ level of brand preference will demonstrate a significant positive correlation with the memory of the brand’s relevant information;*
*Hypothesis 2c: The higher the level of preference for a brand that subjects hold, the better the overall recognition and R rates of positive information, when compared to negative information.*

### Methods

#### Participants

Forty participants (20 males, *M*_age_ = 22.35 years, *SD*_age_ = 1.09) with normal or corrected to normal vision were recruited from universities in Shanghai.

#### Experimental Design

The experiment was a 3 (referential task: high vs. moderate vs. low levels of brand preference) × 2 (information valence: positive vs. negative) within-subjects design experiment. The dependent variables were recognition rates and R/K rates.

#### Procedure

The materials, procedure, and measures were similar to those described in Experiment 1. Please see Appendix B for details. The differences were that subjects were asked to evaluate the degree of preference of shampoo brands as “high,” “moderate,” or “low,” and that the adjectives were divided into six groups.

### Results

This study used SPSS 23.0 to analyze data from the questionnaire, recognition rates, and R/K rates.

#### Manipulation Checks

A within-subject, one-way ANOVA on preference was performed. With referential task as the independent variable, significant main effect of different brands was observed [*F*(2,119) = 179.19, *p* < 0.001, η^2^ = 0.75]. Further pair-wise comparisons showed that the three brands demonstrated significantly different levels of preference compared with one another (*ps* < 0.001. high preference brand: *M* = 5.80, *SD* = 1.09; moderate preference brand: *M* = 4.33, *SD* = 1.16; low preference brand: *M* = 1.53, *SD* = 0.78).

Moreover, a repeated measures, one-way ANOVA on the recognition rates of new and old words revealed a significant effect of word state [*F*(1,39) = 166.13, *p* < 0.001, η^2^ = 0.81]. In other words, the recognition rate of old words was higher than that of new words, demonstrating an effective manipulation.

#### Recognition Rate

Descriptive statistics of recognition rates and R/K rates of different referential task and different information valence are presented in Table 3. A 3 (referential task: high vs. moderate vs. low levels of self-preference brands) × 2 (information valence: positive vs. negative) repeated measures ANOVA on recognition rates revealed a significant main effect of information valence [*F*(1,39) = 10.51, *p* = 0.002, η^2^ = 0.21], indicating that subjects had better recognition of positive words than negative words. There was a non-significant main effect of referential task [*F*(2,78) = 0.85, *p* = 0.43, η^2^ = 0.02].

**Table 3:** The M (SD) of the recognition rate and R/K rate of different referential tasks and information valence.

Memory	Information
indexes	valence	Referential tasks		
		High-	Moderate-	Low-
		preference	preference	preference
		brands	brands	brands
Recognition rate	Positive	0.72 (0.27)_a1_	0.65 (0.25)	0.61 (0.23)_2_
	Negative	0.53 (0.23)_b1_	0.62 (0.28)_2_	0.60 (0.18)_2_
	Mean	0.62 (0.20)	0.64 (0.18)	0.60 (0.19)
R rate	Positive	0.39 (0.24)_a1_	0.34 (0.19)_a_	0.28 (0.20)_2_
	Negative	0.24 (0.16)_b_	0.25 (0.18)_b_	0.24 (0.17)
	Mean	0.28 (0.19)	0.26 (0.17)	0.24 (0.17)
K rate	Positive	0.37 (0.28)	0.37 (0.18)	0.36 (0.18)
	Negative	0.32 (0.20)	0.39 (0.30)	0.38 (0.18)
	Mean	0.34 (0.18)	0.37 (0.17)	0.37 (0.16)

*a and b in each column, 1 and 2 in each line had a significant difference, *p* < 0.05*.

A significant interaction [*F*(2,78) = 4.06, *p* = 0.02, η^2^ = 0.09] was revealed. When referring to high-preference brands, there was a significant difference between the recognition rate of positive words and negative words [*F*(1,39) = 15.27, *p* < 0.001, η^2^ = 0.28]. In the other two conditions, there was no significant memory difference between positive and negative words [when referring to moderate-preference brands*, F*(1,39) = 0.40, *p* = 0.53, η^2^ = 0.01; when referring to low-preference brands*, F*(1,39) = 0.25, *p* = 0.62, η^2^ = 0.01].

#### R Rate

A 3 (referential task: high vs. moderate vs. low self-preference brands) × 2 (information valence: positive vs. negative) repeated measures ANOVA on R rates also revealed a significant main effect of information valence [*F*(1,34) = 17.56, *p* < 0.001, η^2^ = 0.34], indicating that subjects had better R rates of positive than negative words. There was a non-significant main effect of referential task [*F*(2,68) = 1.76, *p* = 0.18, η^2^ = 0.05].

A significant interaction [*F*(2,68) = 3.96, *p* < 0.05, η^2^ = 0.10] was revealed. When referring to high-preference brands, there was a significant difference between the R rate of positive words and negative words [*F*(1,33) = 17.28, *p* < 0.001, η^2^ = 0.34]. When referring to moderate-preference brands, the R rate of positive words was also significantly better than negative words [*F*(1,33) = 6.90, *p* < 0.05, η^2^ = 0.17]. Specifically, the memory difference between positive and negative words was larger when referring to high-preference brands than moderate-preference brands. However, when referring to low-preference brands, there was no significant memory difference between positive and negative words [*F*(1,33) = 2.89, *p* = 0.10, η^2^ = 0.08].

#### K Rate

A 3 (referential task: high vs. moderate vs. low self-preference brands) × 2 (information valence: positive vs. negative) repeated measures ANOVA on K rates found no significant main effects of referential task and information valence [*F*_referentialtask_ (3,117) = 0.55, *p*_referentialtask_> 0.05, η^2^_referentialtask_ = 0.01, *F*_informationvalence_ (1,33) = 0.08, *p*_informationvalence_> 0.05, η^2^_informationvalence_ = 0.002]. The interaction between referential task and information valence was also non-significant [*F*(3,114) = 0.55, *p* > 0.05, η^2^ = 0.01].

#### Correlations Between Brand Preference and Memory

Correlations between the degree of brand preference, recognition rate, R rate, and K rate were calculated, and are reported in Table 4. The degree of brand preference demonstrated significant positive correlations with the overall recognition rate (*r* = 0.18, *p* < 0.05) and R rate (*r* = 0.21, *p* < 0.05), indicating that the higher the brand preference, the higher the overall recognition and R rates. The degree of brand preference also had significant positive correlations with the recognition rate of positive words (*r* = 0.26, *p* < 0.01) and the R rate of positive words (*r* = 0.30, *p* < 0.01), indicating that the higher the brand preference, the higher the recognition and R rates of positive words. The K rate of positive words and the memory of negative words were not significantly related to brand preference.

**Table 4:** Correlations (*r*) among preference, recognition rate, R rate, and K rate of positive and negative words.

	Recognition	R rate	K rate	Recognition	Recognition	R rate	R rate	K rate	K rate
	rate of all	of all	of all	rate of PW	rate of NW	of PW	of NW	of PW	of NW
Preference	0.18^∗^	0.21^∗^	-0.01	0.26^∗∗^	0.01	0.30^∗∗^	0.06	0.03	-0.04

*PW, positive words; NW, negative words. ^∗∗∗^*p* < 0.001, ^∗∗^*p* < 0.01, ^∗^*p* < 0.05, ^†^*p* < 0.10*.

### Discussion

ANOVA revealed no significant main effect of referential task on the overall recognition rate, R rate, or K rate, indicating no difference in the memory of high-, moderate- and low-preference brands, and the degree of brand preference did not appear to have an impact on memory, failing to support hypothesis 2a. However, correlation analysis showed that the degree of brand preference demonstrated significant positive correlations with overall recognition and R rates, supporting hypothesis 2b. In general, the impact of brand preference on memory was partly supported, fitting with the theory of the self-expansion model that people will include brands they prefer into the self-concept, resulting in more complex processing. Simultaneously, according to the self-expansion model, the low product involvement of shampoo might be the cause of the current results. In other words, the weak relationship of shampoo with the self may have led to the non-significant memory differences among three brands. Since highly involved products, such as laptops might support hypothesis 2a, Experiment 3 added product involvement as an independent variable and predicted different memory effects of high-involvement products with different brand preference.

Experiment 2 also found that higher brand preference led to larger memory differences between positive and negative information. ANOVA results revealed that recognition rate, only when referring to a high-preference brand, would result in memory differences between positive and negative words. While for the R rate, this effect was seen when referring to both high- and moderate-preference brands, with the memory difference being larger when referring to highly preferred brand when compared to a moderately preferred brand. Additionally, correlation results also revealed that brand preference demonstrated significant positive correlations with recognition and R rates of positive words. These results mean that the memory difference between positive and negative information will likely increase along with an increase of brand preference, fitting with self-schema theory and the mnemic neglect model. Since the R rate was more sensitive than recognition rate, a stronger difference was noted. Therefore, similar to Experiment 1, hypothesis 2c of Experiment 2 was also supported.

## Experiment 3: The Effect of Product Involvement, Brand Preference, and Valence of Relevant Information on Memory

This study sought to determine the impact of product involvement, brand preference, and information valence on brand-related information memory.

### Experimental Hypotheses

According to Chen (2007), product involvement influences the connection between products and consumers, meaning that higher involvement leads to a closer connection. Based upon the self-expansion model (Aron and Aron, 1986), since consumers demonstrate more elaborate processing of high-involvement products, the products are more likely to be deeply included into self-concept. Therefore, the current study predicted that the recognition and R rates of high-involvement products would be better than low-involvement products. Furthermore, when products were classified as high-involvement, brand preference will demonstrate a significant influence on memory under this condition. Conversely, low-involvement products would be more weakly included into the self, thus the influence of brand preference on memory will be weakened under this condition. The effect of information valence would also only be significant when both product involvement and brand preference were high according to self-schema theory. Based on the results of Experiment 2, a further hypothesis was raised, that both the memory differences between high-preference and low-preference brands, as well as the differences between positive and negative words of high-preference brands may only appear when referring to high-involvement products (which undergo better information processing). As such, when referring to low-involvement products, the impact of brand preference would be weakened because of weaker information processing.

In summary, the hypotheses of Experiment 3 include:

(1) Hypotheses about the influence of brand preference on memory:
          *Hypothesis 3a: The higher preference subjects had for a brand, the better overall recognition and R rates of its relevant information;*
          *Hypothesis 3b: Subjects’ brand preference will demonstrate a significant positive correlation with the memory of the product’s relevant information;*
(2) Hypotheses about the influence of product-involvement on memory:
          *Hypothesis 3c: The higher the level of involvement subjects had with the product, the better overall recognition and R rates of the product’s relevant information;*
          *Hypothesis 3d: Subjects’ involvement with product will demonstrate a significant positive correlation with the memory of the product’s relevant information;*
(3) Hypotheses about the interaction between brand preference and product-involvement:
          *Hypothesis 3e: The overall recognition and R rates of high-preference brands will be significantly better than that of low-preference brands, but only in high-involvement products. Conversely, these memory differences will not be observed when product involvement was low;*
(4) Hypotheses about the interaction of brand preference, product-involvement, and information valence:
          *Hypothesis 3f_1_: When both product involvement and brand preference were high, the recognition and R rates of positive information will be significantly better than those of negative information.*
          *Hypothesis 3f_2_: When product involvement was high and brand preference was low, no significant memory differences between positive and negative information will be observed.*
          *Hypothesis 3f_3_: When the product involvement was low, no significant memory differences between positive and negative information will be observed.*

### Methods

#### Participants

Sixty-six participants (35 males, *M*_age_ = 23.03 years, *SD*_age_ = 2.47) with normal or corrected to normal vision were recruited from universities in Shanghai.

#### Experimental Design

The experiment was a 2 (referential task: high- vs. low-preference brands) × 2 (product involvement: high vs. low) × 2 (information valence: positive vs. negative) mixed design experiment. The between-subject variable was product involvement and the others variables were the within-subject variables. The dependent variables were recognition rates and R/K rates.

#### Procedure

The materials, procedure, and measures were similar to those reported in Experiments 1 and 2. Please see Appendix C for details. The difference was that Experiment 3 used two different types of products, one with high-involvement (laptops) and one with low-involvement (shower gels). Subjects were asked to complete a Personal Involvement Inventory (PII; Zaichkowsky, 1985; Polegato and Zaichkowsky, 1994) to obtain involvement scores and ensure that the level of involvement for laptops was higher than that of shower gels. The PII was developed to assess levels of involvement for products, and was shown to have good reliability and validity (Zaichkowsky, 1985). In this study, the Cronbach’s α of PII is 0.97. Participants were asked to evaluate the product using a questionnaire with 10 pairs of attributes such as *important–unimportant*, each evaluated on a seven-point scale (e.g., 1 = *very important*, 7 = *very unimportant*). The product with lower total points would be the higher involved product. In both the laptop and shower gel group, the half of the subjects were male and the other half were female. Participants were asked to evaluate the degree of preference for high- and low-preference brands, with adjectives being divided into four groups.

### Results

SPSS 23.0 was used to analyze the questionnaire, recognition rates, R rates, and K rates.

#### Manipulation Checks

A higher PII score indicates lower product involvement. A repeated measures, one-way ANOVA on involvement scores, with two involvement levels (one high and one low) set as the independent variable, showed a significant main effect, *F*(1,65) = 684.32, *p* < 0.001, η^2^ = 0.91, indicating that the PII could distinguish two different involvement groups. The high-involvement group (*M* = 2.07, *SD* = 0.64) was the laptop group, and the low-involvement group (*M* = 5.59, *SD* = 0.44) was the shower gel group. This manipulation check showed that the two materials used in Experiment 3 had significantly different levels of involvement, meeting the experimental demand.

Brand preference scores were then analyzed. Subjects’ degree of preference for a brand with high involvement and high preference was 6.03 (*SD* = 0.85), for a brand with high involvement and low preference was 2.00 (*SD* = 1.02), for brand with low involvement and high preference was 5.47 (*SD* = 1.08), and for brand with low involvement and low preference was 2.09 (*SD* = 1.12). A 2 (referential task: high- vs. low-preference brands) × 2 (product involvement: high vs. low) repeated measures ANOVA on preference scores revealed significant main effects of referential task of brand preference [*F*(1,64) = 438.66, *p* < 0.001, η^2^ = 0.87] and product involvement [*F*(1,64) = 1846.15, *p* < 0.001, η^2^ = 0.90]. The interaction was not significant, *F*(1,64) = 3.31,*p* = 0.07, η^2^ = 0.05. Therefore, subjects demonstrated different degrees of preference for brands with different levels of involvement, meeting the experimental demand.

Moreover, a repeated measures, one-way ANOVA on the recognition rates of new words and old words revealed a significant effect of word state [*F*(1,65) = 581.36, *p* < 0.001, η^2^ = 0.91], indicating an effective manipulation of old and new words.

#### Recognition Rate

Descriptive statistics of recognition, R, and K rates of different referential task, different levels of product involvement, and different informational valence are presented in Table 5. A 2 (referential task: high- vs. low-preference brands) × 2 (product involvement: high vs. low) × 2 (information valence: positive vs. negative) repeated measures ANOVA on recognition rates revealed a significant main effect of referential task [*F*(1,64) = 6.07, *p* = 0.02, η^2^ = 0.09], indicating that the memory of a highly preferred brand was better than lowly preferred brand. A significant main effect of information valence [*F*(1,64) = 6.62, *p* = 0.01, η^2^ = 0.09] was also observed, indicating that the memory of positive words (*M* = 0.34, *SD* = 0.10) was better than that of negative words (*M* = 0.31, *SD* = 0.10). No significant main effect of product involvement was found, *F*(1,64) = 2.66, *p* = 0.11, η^2^ = 0.04.

**Table 5:** The M (SD) of the recognition, R, and K rates of referential tasks, product involvement, and information valence.

Memory indexes	Information valence	Product involvement level			
		High		Low	
		High-preference	Low-preference	High-preference	Low-preference
		brands	brands	brands	brands
Recognition rate	Positive	0.34 (0.09)	0.30 (0.11)	0.37 (0.09)_a_	0.35 (0.10)
	Negative	0.32 (0.10)	0.29 (0.10)	0.32 (0.11)_b_	0.33 (0.08)
	Mean	0.33 (0.10)_1_	0.29 (0.10)_2_	0.34 (0.10)	0.34 (0.09)
R rate	Positive	0.26 (0.12)_a_	0.22 (0.13)_a_	0.23 (0.13)_a_	0.22 (0.11)
	Negative	0.22 (0.12)_b_	0.19 (0.10)_b_	0.19 (0.13)_b_	0.21 (0.12)
	Mean	0.24 (0.12)_1_	0.21 (0.12)_2_	0.21 (0.13)	0.22 (0.12)
K rate	Positive	0.08 (0.08)	0.08 (0.09)	0.13 (0.12)	0.13 (0.11)
	Negative	0.10 (0.08)	0.09 (0.07)	0.13 (0.13)	0.12 (0.12)
	Mean	0.09 (0.08)	0.09 (0.08)	0.13 (0.12)	0.12 (0.11)

_a_*and _*b*_ in each column, _*1*_ and _*2*_ in each line had a significant difference, *p* < 0.05*.

The interaction between brand preference and product involvement was marginally significant [*F*(1,64) = 3.17, *p* = 0.08, η^2^ = 0.05]. For further simple effect analyses, we fixed the involvement and analyzed brand preference. When involvement was high, the recognition rate of high-preference brands was significantly better than low-preference brands [*F*(1,64) = 8.25, *p* = 0.006, η^2^ = 0.11]; when involvement was low, there was no significant memory difference between high- and low-preference brands [*F*(1,64) = 0.26, *p* = 0.61, η^2^ = 0.004]. Additionally, no significant interactions between product involvement and information valence [*F*(1,64) = 0.29, *p* = 0.59, η^2^ = 0.01], between brand preference and information valence [*F*(1,64) = 1.77, *p* = 0.19, η^2^ = 0.03], or among the three of these variables [*F*(1,64) = 0.97, *p* = 0.33, η^2^ = 0.02] were observed.

#### R Rate

A 2 (referential task: high- vs. low-preference brands) × 2 (product involvement: high vs. low) × 2 (information valence: positive vs. negative) repeated measures ANOVA on R rates revealed a marginally significant main effect of referential task [*F*(1,64) = 3.30, *p* = 0.07, η^2^ = 0.05], indicating that the memory of high preference brands was better than that of low preference brands. Additionally, a significant main effect of information valence [*F*(1,64) = 11.02, *p* = 0.001, η^2^ = 0.15] was observed, indicating that the memory of positive words (*M* = 0.23, *SD* = 0.12) was better than that of negative words (*M* = 0.20, *SD* = 0.12). However, no significant main effect of product involvement was found [*F*(1,64) = 0.12, *p* = 0.73, η^2^ = 0.002].

The interaction between brand preference and product involvement was significant [*F*(1,64) = 4.98, *p* < 0.05, η^2^ = 0.07]. For further simple effect analyses, we fixed involvement and analyzed brand preference. When involvement was high, the R rate of high-preference brands was significantly better than that of low-preference brands [*F*(1,64) = 7.52, *p* = 0.008, η^2^ = 0.11]; while, when involvement was low, there was no significant memory difference between high- and low-preference brands [*F*(1,64) = 0.09, *p* = 0.76, η^2^ = 0.001]. The interaction between brand preference and information valence was marginally significant [*F*(1,64) = 2.85, *p* = 0.096, η^2^ = 0.04]. For further simple effect analyses, we fixed the brand preference and analyzed information valence. Although the memory of positive words was always better than negative words in both high- and low-preference brands, the memory difference between positive and negative words of high-preference brands showed a higher level of significance [*F*(1,64) = 12.96, *p* = 0.001, η^2^ = 0.17] than that of low-preference brands [*F*(1,64) = 3.98, *p* = 0.05, η^2^ = 0.06]. Additionally, no significant interactions between product involvement and information valence [*F*(1,64) = 0.22, *p* = 0.64, η^2^ = 0.003], or among the three independent variables [*F*(1,64) = 1.03, *p* = 0.31, η^2^ = 0.02] were found.

#### K Rate

A 2 (referential task: high- vs. low-preference brands) × 2 (product involvement: high vs. low) × 2 (information valence: positive vs. negative) repeated measures ANOVA on K rates found no significant main effects of referential task [*F*(1,64) = 1.92, *p* = 0.17, η^2^ = 0.03], product involvement [*F*(1,64) = 2.81, *p* = 0.10, η^2^ = 0.04], or information valence [*F*(1,64) = 0.50, *p* = 0.49, η^2^ = 0.01]. Further, the interactions between referential task and product involvement [*F*(1,64) = 0.26, *p* = 0.61, η^2^ = 0.004], between referential task and information valence [*F*(1,64) = 0.004, *p* > 0.05, η^2^ = 0.000], between product involvement and information valence [*F*(1,64) = 1.75, *p* = 0.19, η^2^ = 0.03], and among the three variables [*F*(1,64) = 0.001, *p* = 0.98, η^2^ = 0.000] were all found to be non-significant.

#### Correlations Between Brand Preference, Product Involvement, and Memory

Since this experiment was a mixed design, with two groups of participants judging high-involvement laptops and low-involvement shower gels as materials, respectively, correlation analyses were separately computed. Correlations among the degree of laptop brand preference, product involvement, recognition rate, R rate, and K rate were compared and are reported in Table 6. Product involvement was negatively related to the overall R rate (*r* = -0.31, *p* < 0.05) and positively related to the overall K rate (*r* = 0.41, *p* < 0.01), indicating that, in high-involvement laptops, the higher the level of involvement people had with the product (lower PII score meant higher involvement), the higher R rate would be, and the lower K rate would be. Product involvement was also negatively related with the R rate of positive words (*r* = -0.32, *p* < 0.05) and negative words (*r* = -0.25, *p* < 0.10), positively related with the K rate of positive words (*r* = 0.40, *p* < 0.01) and negative words (*r* = 0.34, *p* < 0.01), indicating that the higher the involvement level toward laptops, the higher R rate of positive and negative words would be, and the lower the K rate of these words would be. The degree of brand preference had significant positive correlations with the overall recognition rate (*r* = 0.29, *p* < 0.05) and recognition rate of negative words (*r* = 0.28, *p* < 0.05).

**Table 6:** Correlations (*r*) among brand preference, product involvement, recognition rate, R rate, and K rate of positive and negative words describing laptops.

	Recognition	R rate	K rate	Recognition	Recognition	R rate	R rate	K rate	K rate
	rate of all	of all	of all	rate of PW	rate of NW	of PW	of NW	of PW	of NW
Product involvement	-0.04	-0.31*	0.41**	-0.07	-0.01	-0.32*	-0.25^†^	0.40**	0.34**
Preference	0.29*	0.17	0.09	0.21	0.28*	0.16	0.16	0.03	0.14

*PW, positive words; NW, negative words. ^∗∗∗^*p* < 0.001, ^∗∗^*p* < 0.01, ^∗^*p* < 0.05, ^†^*p* < 0.10*.

Correlations among the degree of shower gel brand preference, product involvement, recognition rate, R rate, and K rate were compared and are reported in Table 7. Product involvement was negatively related with the overall K rate (*r* = -0.26, *p* < 0.05), indicating that, in the low-involvement shower gel brands, the higher involvement people had toward the product, the higher the K rate would be. Product involvement was also marginally related with the recognition rate of negative words (*r* = -0.20, *p* < 0.10), and was negatively related to the K rate of both positive words (*r* = -0.24, *p* < 0.05) and negative words (*r* = -0.25, *p* < 0.05), indicating that the higher involvement people had toward shower gels, the higher the recognition rate of negative words, and the higher K rates of positive and negative words would be. The degree of brand preference demonstrated a non-significant correlation with overall recognition rate, R rate, and K rate, but it was marginally related to the recognition rate of positive words (*r* = 0.20, *p* < 0.10).

**Table 7:** Correlations (*r*) among brand preference, product involvement, recognition rate, R rate, and K rate of positive and negative words describing shower gels.

	Recognition	R rate	K rate	Recognition	Recognition	R rate	R rate	K rate	K rate
	rate of all	of all	of all	rate of PW	rate of NW	of PW	of NW	of PW	of NW
Product involvement	-0.17	0.14	-0.26*	-0.10	-0.20^†^	0.16	0.09	-0.24*	-0.25*
Preference	0.07	-0.07	0.11	0.20^†^	-0.08	0.03	-0.15	0.13	0.09

*PW, positive words; NW, negative words. ^∗∗∗^*p* < 0.001, ^∗∗^*p* < 0.01, ^∗^*p* < 0.05, ^†^*p* < 0.10*.

### Discussion

ANOVAs revealed that the overall recognition and R rates of high-preference brands were better than that of low-preference brands, supporting hypothesis 3a. However, product involvement did not demonstrate significant main effects, failing to support hypothesis 3c. The significant interaction between brand preference and product involvement showed that memory of high-preference brands was better than that of low-preference brands, but only when the product had high involvement, supporting hypothesis 3e. Finally, the memory difference displayed in R rate between positive and negative words was larger in the high-preference condition than in low-preference condition, partly supporting hypotheses 3f_1_ and 3f_2_.

Correlation analyses showed that the preference level for laptops was positively related with recognition rate, and that the preference level for shower gels was marginally related with the recognition rate of positive words, supporting hypothesis 3b. Additionally, the preference for shower gel did not demonstrate significant correlations with overall recognition rate or R rate, again supporting hypothesis 3e. In the laptop group, higher involvement was associated with a better R rate and a worse K rate; conversely, in the shower gel group, higher involvement was associated with a better K rate, supporting hypothesis 3d.

Experiment 1 found that the overall memory of self-preferred brands was worse than that of other-preferred brand, and, Experiment 2 found that, although preference demonstrated positive correlations with recognition and R rates, it did not have a significant main effect, both of these results failing to support the referential effect of preferred brands. The materials used by the former two studies were shampoo brands that were familiar to everyone. However, subjects’ levels of involvement with the shampoos were different, possibly contributing to different levels of self-inclusion. When consumers had a close connection with a product, the results of these two experiments suggested a deeper inclusion to the self-concept.

Given this information, Experiment 3 added the variable of product involvement. Two groups of participants were selected by questionnaire, which were a high-involvement laptop group and a low-involvement shower gel group. Results showed that, in overall recognition and R rates, there were significant memory differences between high-preference brands and low-preference brands in the laptop group (i.e., the memory of high-preference brands was better). However, in the shower gel group, these memory differences disappeared, not only providing support for hypothesis 3e, but also explaining why hypothesis 2a of Experiment 2, which was that higher preference would lead to better memory, was not supported.

On overall recognition and R rates, information valence had significant main effects, indicating that the memory of positive information was always better than that of negative information. Moreover, the interaction of this variable with referential task was marginally significant on R rate, showing that, despite the memory advantage of positive information in both high- and low-preference conditions, the advantage was far more significant in the high-preference condition. Therefore, hypotheses 3f_1_ and 3f_2_ was partially supported. However, the impact of involvement in hypotheses 3f_1_, 3f_2_, 3f_3_, and 3c was not observed, which may be attributed to other unknown factors playing a part in influencing memory. Product involvement might not have an independent impact on memory of brand-related information, but rather, it could work together with brand preference, indicating that the formation of referential memory is a complicated process.

Based on the prior experiments, Experiment 3 additionally found that, in the laptop group, higher involvement was associated with a lower K rate, but in the shower gel group, higher involvement was associated with a higher K rate. These results suggest that individuals can easily form a precise and detailed memory for high-involvement products, but only a vague and integral memory for low-involvement products.

## General Discussion

### Memory Difference Between Positive and Negative Brand-Related Information

The memory difference between positive and negative information was only shown in recognition and R rates, which conforms to prior research suggesting that the SRE was more impactful in recognition and R responses (Liu and Zhu, 2002). This is because R responses contained a self-awareness element, and were closely linked to the self-concept, while K responses were based only on semantics.

Experiment 1 revealed that the memory of positive words of self-preferred brands was better than that of negative words, and that there was no significant memory difference between words with different valence of other-preferred brands. Moreover, subjects’ memory of negative words of self-preferred brands was worse than the memory of negative words of other-preferred brands. Experiment 2 revealed that, when referring to highly and moderately preferred brands, the memory of positive words was significantly better than negative words, and that the memory difference was more significant when referring to high-preference brands. When referring to low-preference brands, there was no memory difference between positive and negative words. The results on R rate in Experiment 3 revealed that, although the memory of positive words was always better than negative words, their difference was more significant in the high-preference condition than in the low-preference condition. All of the results reported are in agreement with the prior conclusion that positive and negative words had significant memory differences in the SRE. The important effect of brand preference on the memory of positive and negative words was supported, indicating that the higher preference subjects had for a brand, the more relevant positive information they could remember.

According to self-schema theory, consumers will incorporate the positive images of their preferred brands into the self-concept, and be loyal to the brand, thus reflecting the positive images of themselves. Therefore, brand-related positive information can receive more complex processing, resulting in better memory. Moreover, the higher the individual’s preference for the brand, the more likely he/she will internalize the relevant positive information and enhance his/her loyalty. Hence, the recognition and R rates of positive information will be much higher than those of negative words when the brand preference is higher, and when the product involvement is higher. The mnemic neglect model (Chen and Zhao, 2009; Newman et al., 2009) has pointed out that people have a self-protection mechanism which automatically ignores negative information that threatens the self. Ahluwalia et al. (2000) also found that brand loyalty could weaken the harmful effects caused by negative incidents, and that consumers with high brand loyalty would resist negative information about the brand. As a result, it is easier for subjects to ignore negative information of their preferred brands, weakening memory processing.

Many prior studies (e.g., Kuiper and Derry, 1982) have found that the memory of positive trait adjectives is better than negative ones within the context of the SRE. However, no researchers have studied the memory difference of information valence under brand-referential conditions. This study added information valence as an independent variable while analyzing the effect of brand preference and product involvement on memory, aiming to determine whether the memory difference that appears in the SRE would also exist in brand-referential conditions. The results supported the hypothesis. Subjects’ memory of positive information of preferred brands was always better than the memory for negative information, and the memory advantage of positive information was more significant when brand preference and product involvement was higher. This is in accordance with self-schema theory, in that subjects would tend to include positive information of preferred brands into schemata. Similar to studies in the self-domain, for instance, self-esteem is one kind of self-schemata that is suggested to influence the memory of positive and negative words, with subjects reporting higher self-esteem demonstrating a memory preference for positive adjectives (Tian and Zhang, 2008; Yang et al., 2012). Therefore, the self includes the positive information of the self, others, and some objects into self-schemata through a cognitive process, promoting elaborate processing, and resulting in a memory advantage.

### The Effect of Brand Preference and Product Involvement on Memory

Similar to previous topics, all of the results of the effect of brand preference and product involvement were only reflected in recognition and R rates. The results of Experiments 2 and 3 showed that preference demonstrated significant positive correlations with recognition and R rates, indicating that higher brand preference might lead to better memory. In the laptop group, product involvement was negatively related with R rate and positively related with K rate; in the shower gel group, involvement was negatively related with K rate. It was shown that, in high-involvement products, higher involvement made people “remember” more and “know” less, but in low-involvement products, the overall memory was worse because of the low involvement (i.e., “connection”) with the product. Further, in the case of the current results, higher involvement made people “know” more and memorize better. In addition to the results discussed above, preference and product involvement demonstrated a significant interaction (i.e., when products had high involvement, higher brand preference leaded to better memory and was positively correlated with recognition rate; when products had low involvement, brand preference had no effect on memory and no correlation with memory). The overall results showed a preferred brand reference effect.

According to the self-expansion model (Aron and Aron, 1986; Aron et al., 1991), subjects have a motivation to include other people into the self. Jia and Shi (2012) put forward that the expansion can be acquired, not only from intimate relationships, but also from other sources, such as new events or familiar objects. Zhou et al. (2012) his also found that subjects’ free recall scores of self-owned objects were significantly higher than objects owned by others, showing the ownership effect (i.e., that the mere virtual ownership relation determined by pronouns, such as “my” and “his” impact memory). As a result, subjects can indeed form the schemata of preferred brands by including them into the self-concept, gaining a memory advantage similar to that found with the SRE.

Furthermore, the intimacy between self and others was found to influence the self-referential memory advantage (Symons and Johnson, 1997; Zhou and Su, 2008; Xiong et al., 2012). According to the self-expansion model, the memory of highly intimate others will be of higher quality because they are more deeply included into the self. During memory formation, brand preference and product involvement have a similar impact to intimacy. The higher involvement people have with a product and the higher preference they have for a brand, the deeper the concepts of brand/product will be included into the self. Thus, both of these elements will lead to better memory.

On this theoretical basis, the current study provides further evidence to support the impacts of brand preference and product involvement, as well as their interaction. Specifically, higher involvement and higher preference was associated with better memory. The effect of brand preference was more significant when product involvement was high, and it was weakened when product involvement was low. The non-significant main effect of product involvement in Experiment 3 also gave credence to the notion that consumers’ cognition of brands was not completely fair, and that the formation of brand preference is a complicated process. Xu (2012) pointed out that consumers’ perceived risk could also influence the effect of involvement on brand preference. To some extent, this conclusion can explain why the effect of brand preference on memory was not observed in Experiment 1. The reason the memory of self-preferred brands was not better than other-preferred brands might be due to brand preference being affected by many other factors, such as product involvement.

According to the self-expansion model, previous research has paid most attention to subjects’ tendency to include intimate others into the self. This study moved forward a single step to provide supporting evidence that subjects would also try to include preferred brands into the self. During the process of forming a brand preference, consumers take the concept, identity, and even the image of the brands into the self. The more loyal consumers are to the brands, the deeper they will be incorporated into identity, the more the brand-related concept will overlap with the self-concept, and the more tags will be attached to a memory associated with the brand (making recall easier). The existing SRE studies have all verified that memories of self-related information are better than memories of other types of information. This is purportedly due to individuals having enhanced schemata of the self and intimate others (Li and Meng, 2001). These enhanced schemata include both elaborate and organizational processing. Similarly, people would also demonstrate this same processing advantage in brands with higher preference and higher involvement levels. The correlation between the self and preferred brands with high involvement is quite large, making it easier for consumers to purchase such brands under the same conditions. Self-image consistency theory (Dolich, 1969) has also suggested that consumers choose and prefer brands that are consistent with their image, and are loyal to brands to enhance their self-concepts. This study supports the idea that people will also incorporate preferred brands into the self, causing the effect of brand preference.

### Limitations and Future Directions

Based on the paradigm of the SRE, this study presents preliminary evidence of the application of the SRE in brand preference, with different levels of product involvement. However, the current study did contain some limitations.

First, Qi and Zhu (2002) added “Familiar” (F) and “Guess” (G) responses into the SRE research paradigm. Through the course of study, it was found that there was no significant difference between F responses and K responses, so R/K/G responses were used. This study used the R/K SRE paradigm, with subjects only able to choose R or K responses. Subsequent studies may consider a more rigorous R/K/G paradigm.

Second, in the memory reference effect of brand preference, the influencing factors are likely far more complex than only brand preference and product involvement. For future research, it is suggested to explore the brand reference effect further. In a study by Zheng (2012), a new variable of emotion was introduced under the arbitrary coding self-reference paradigm. In brand consumption, emotion is an important variable and cannot be ignored. Researchers can consider introducing positive and negative emotions in the future, and choosing different brand products for further study. Xie (2013) also found that boys were more likely than girls to display high scores of memory when referring to fathers, but lower scores than girls when referring to mothers, providing evidence for the impact of gender, which should be further explored. In addition, the stereotypes held regarding high-preference and low-preference brands may also play a role in relevant information memory. In conclusion, this study has discussed the influence of brand on memory for the first time. However, there are likely many other influential factors that have not been considered, requiring the need for further exploration and validation.

On a final note, this study was limited by only using the SRE paradigm to perform experimental research. Future research can use methods of cognitive neuroscience, such as ERPs and fMRI, to study the cognitive process of brand information memory, further broadening the research scope of the reference effect. Moreover, new methods of “big data” collection can be adopted to gather vast quantities of information regarding brand preference and cognition on the Internet, (e.g., positive and negative evaluation of preferred brands), and related data analysis can be performed to collect more empirical evidence for this topic.

## Conclusion

The current series of experiments has provided evidence that the higher the degree of brand preference, the better the memory of relevant positive information when compared to negative information. Additionally, brand preference and product involvement both affect brand-related memory and have an interactive effect. Furthermore, when product involvement was high, brand preference was elevated, resulting in a better the memory effect. However, when product involvement was low, there was no difference between the memory of high-preference and low-preference brands.

## Ethics Statement

This study was carried out in accordance with the recommendations of “University Committee on Human Research Protection of East China Normal University” with written informed consent from all subjects. All subjects gave written informed consent in accordance with the Declaration of Helsinki. The protocol was approved by the “University Committee on Human Research Protection of East China Normal University.”

## Author Contributions

RF and WM were responsible for the overall design, implementation, and data analysis of the three experiments. RL and XL were responsible for part of the thesis writing and data analysis. ZZ and JX were responsible for the overall inspection and correction of manuscript. MZ, TQ, and CQ were responsible for the implementation of Experiments 3, 1, and 2, respectively.

## Conflict of Interest Statement

The authors declare that the research was conducted in the absence of any commercial or financial relationships that could be construed as a potential conflict of interest.
