# Supplementary material for: How Preferred Brands Relate to the Self: The Effect of Brand Preference, Product Involvement, and Information Valence on Brand-Related Memory
Source: Front Psychol. 2019 Apr 26;10:783. doi: 10.3389/fpsyg.2019.00783 (PMC6499029; doi:10.3389/fpsyg.2019.00783)
Supplement: Supplementary file 1 [file Table_1.DOCX]

**Appendix A: Materials of Experiment 1**

**Questionnaire**

**Questionnaire about shampoo brand preference.**

Thank you for participating in this survey! All answers to this questionnaire will be kept strictly confidential and the results will be a conclusive report that will not reveal any information about you. The results of this study will only be used for academic research and will not be used for any commercial purposes. This survey is not a test, and there are no “right” or “wrong” answers. The effectiveness of the research results depends primarily on whether you reflect your actual opinion. Therefore, we hope that you can express your opinions very carefully, and answer every question, which is very important for our study.

Gender: _________

Age: ___________

The following are the shampoo brands on the market:

**Head & Shoulders, Pantene, Clear, Rejoice, Clairol, Bawang,**

**Vidal Sassoon, Dove, Syoss, Revlon, Aquair**

Please select your favorite shampoo brand from the above shampoo brands, fill in the blank below, and complete the following questionnaire.

__________________

1. I like this shampoo brand.

Strongly Disagree 1 2 3 4 5 6 7 Strongly Agree

2. I am familiar with this brand.

Strongly Disagree 1 2 3 4 5 6 7 Strongly Agree

(Instructions: The previous subject did not choose the same brand as you chose. Please guess his/her favorite shampoo brand, fill in the blank below, and complete the following questionnaire.

__________________

3. I like this shampoo brand.

Strongly Disagree 1 2 3 4 5 6 7 Strongly Agree

4. I am familiar with this brand.

Strongly Disagree 1 2 3 4 5 6 7 Strongly Agree

**Brand Adjectives**

| Table A  *The list of band adjectives in Experiment 1* | | | | |
| --- | --- | --- | --- | --- |
|  | Group 1 | Group 2 | Group 3 | Group 4 |
| 1 | relieved | hygienic | maintaining | necessary |
| 2 | famous | attractive | powerful | nourished |
| 3 | unique | long lasting | frequently-used | profitable |
| 4 | excellent | aromatic | mild | smooth |
| 5 | transcendent | desirable | successful | mature |
| 6 | easeful | comfortable | preferred | economic |
| 7 | outstanding | innovative | pure | unadulterated |
| 8 | reassuring | money-saving | superb | balmy |
| 9 | top-level | moving | prosperous | sweet-scented |
| 10 | silky | fresh | wholesome | gentle |
| 11 | abundance | popular | economical | fragrant |
| 12 | pretty | elegant | cheap | durable |
| 13 | noble | efficient | reasonable | fair |
| 14 | beautiful | environmental | gorgeous | healthy |
| 15 | bleak | dim | dirty | scorched |
| 16 | dangerous | clumsy | vulgar | untruthful |
| 17 | insufficient | inconvenient | illegal | undesirable |
| 18 | inferior | lawless | casual | tacky |
| 19 | incomplete | dismal | cursory | adulterate |
| 20 | decaying | defaulted | failing | luxurious |
| 21 | ugly | smug | greasy | pungent |
| 22 | poor | damaged | mediocre | barren |
| 23 | shoddy | coarse | low-grade | degraded |
| 24 | bad | out-of-date | tired | wasteful |
| 25 | boring | restless | queasy | illicit |
| 26 | dull | withered and yellow | bald | deceptive |
| 27 | painstaking | trivial | closed | superficial |
| 28 | embarrassed | arrogant | dark-minded | counterfeit |

**Instructions**

**Learning phase.**

Welcome to our experiment!

This is an adjective evaluation experiment, and you will need to determine whether each word is appropriate for describing a particular object or whether it is a positive or negative word. The experiment starts with a red “+” sign on the computer screen to remind you where to look. Then, you will be presented the specific object of the adjective evaluation, which comes from the questionnaire you filled in before. After that comes the adjective. According to your own perceptions, please quickly determine if the adjective is suitable to describe the particular object by answering “yes” or “no.” If you think the adjective fits the description, press the “f” button for “yes” and press the “j” button for “no” if the adjective does not fit the object. The process to judging the valence of adjectives is similar to this. If you feel the word has a positive meaning, press “f,” if you feel the meaning is negative, press “j.” The adjectives are only presented for a short time, so please pay close attention and make your judgment.

If you understand the above instructions, please sit down. Then put both hands on the keyboard, with the left hand index finger on the “f” key, the right hand index finger on the “j” key, which are the keys required to answer the questions. Remember that the left hand makes the “suitable” judgment, and the right hand makes the “unsuitable” judgment. Further, the left hand makes a “positive” judgment, the right hand makes a “negative” judgment.

When you are ready, press the “1” button to start the exercise. The formal experiment will then begin.

**Instructions**

**Testing phase.**

Welcome back to our experiment!

Next, there will be a series of adjectives appearing on the screen, some appeared in the previous evaluation stage, and some are new words. Please do your best to quickly determine whether these words appeared in the previous evaluation stage. If you think the word has appeared in the evaluation stage, press the “f” button. If you do not believe that they appeared in the previous stage, press the “j” button. For words that you think have appeared in the evaluation stage, make a further judgment about whether you “remember” or “know” the word.

“Remember” means that you not only recognize the word as being in the evaluation phase, but that you also recall some of the elements and details that occurred when you saw the word. “Know” means that you think you have seen the word in the evaluation stage, and it feels familiar, but you cannot recall the specific details. Please press the “q” button for “remember” and press the “p” button for “know.”

Note: Please respond to all of the above decisions as soon as possible!

When you are ready, press the “1” button to start the exercise. The formal experiment will then begin.

**Appendix B: Materials of Experiment 2**

**Questionnaire**

**Questionnaire about shampoo brand preference.**

Thank you for participating in this survey! All answers to this questionnaire will be kept strictly confidential and the results will be a conclusive report that will not reveal any information about you. The results of this study will only be used for academic research and will not be used for any commercial purposes. This survey is not a test, and there are no “right” or “wrong” answers. The effectiveness of the research results depends primarily on whether you reflect your actual opinion. Therefore, we hope that you can express your opinions very carefully, and answer every question, which is very important for our study.

Gender: _________

Age: ___________

The following are the shampoo brands on the market:

**Head & Shoulders, Pantene, Clear, Rejoice, Clairol, Bawang,**

**Vidal Sassoon, Dove, Syoss, Revlon, Aquair**

Please select the shampoo brands for which you have a high, moderate, and low preference from the above shampoo brands. Fill these into the blanks below and complete the following questionnaire. (Note: Do not fill in the blanks with the top three favored brands.)

**My high-preference shampoo brand is__________________.**

1. I like this shampoo brand very much.

Strongly Disagree 1 2 3 4 5 6 7 Strongly Agree

**My moderate-preference shampoo brand is__________________.**

2. I like this shampoo brand very much.

Strongly Disagree 1 2 3 4 5 6 7 Strongly Agree

**My low-preference shampoo brand is__________________.**

3. I like this shampoo brand very much.

Strongly Disagree 1 2 3 4 5 6 7 Strongly Agree

**Brand Adjectives**

| Table B  *The list of adjectives of brand in experiment 2* | | | | | |
| --- | --- | --- | --- | --- | --- |
| Group 1 | Group 2 | Group 3 | Group 4 | Group 5 | Group 6 |
| safe | hygienic | maintaining | necessary | essential | convenient |
| pure | chaste | decent | top-level | moving | sweet-scented |
| noble | beneficial | attractive | efficient | community-minded | reasonable |
| suitable | economical | black | hair-care | attendant | magnificent |
| delicate | graceful | charming | credible | user-friendly | romantic |
| durable | bushy | cheap | flowing | pretty | unadorned |
| reassuring | modern | fashionable | substantial | preferred | comfortable |
| dim | dirty | expansive | silent | profiteering | degenerative |
| smug | vaunting | pungent | provocative | rough | coarse |
| arduous | trivial | superficial | perfunctory | blundering | rancid |
| blithering | sporty | gray | dark | turbid | counterfeit |
| withered and yellow | exaggerated | dingy | wasteful | tired | out-of-date |
| messy | irritating | hair-damaged | luxurious | failing | defaulted |
| lower | vulgar | clumsy | nauseating | dangerous | bifurcated |
| profitable | carefree | desirable | successful | honest | famous |
| aromatic | abundance | popular | responsible | fragrant | restorative |
| caring | glorious | flashy | bright | normative | valuable |
| cost-efficient | healthy | economic | saving | outstanding | clean |
| effective | gorgeous | nourished | environmental | thick | beautiful |
| wholesome | fresh | silky | submissive | mellow | balmy |
| watery | smooth | aromatic | reliant | hair-tonic | excellent |
| illegal | undesirable | incomplete | dismal | doped | adulterate |
| degraded | hair-loss | wicked | restless | absurd | queasy |
| scorched | stuffy | exotic | bald | allergic | dark-minded |
| arrogant | sallow | embarrassed | susceptible | deceptive | hateful |
| boring | unsmooth | fooling | barren | mediocre | defective |
| cumbersome | tacky | scathing | shrinking | speculative | hair-lost |
| unkempt | sparse | greasy | weak | ineffective | untruthful |

**Instructions**

**Learning phase.**

Welcome to our experiment!

This is an adjective evaluation experiment, and you will need to determine whether each word is appropriate for describing a particular object or whether it is a positive or negative word. The experiment starts with a red “+” sign on the computer screen to remind you where to look. Then, you will be presented the specific object of the adjective evaluation, which comes from the questionnaire you filled in before. After that comes the adjective. According to your own perceptions, please quickly determine if the adjective is suitable to describe the particular object by answering “yes” or “no.” If you think the adjective fits the description, press the “f” button for “yes” and press the “j” button for “no” if the adjective does not fit the object. The process to judging the valence of adjectives is similar to this. If you feel the word has a positive meaning, press “f,” if you feel the meaning is negative, press “j.” The adjectives are only presented for a short time, so please pay close attention and make your judgment.

If you understand the above instructions, please sit down. Then put both hands on the keyboard, with the left hand index finger on the “f” key, the right hand index finger on the “j” key, which are the keys required to answer the questions. Remember that the left hand makes the “suitable” judgment, and the right hand makes the “unsuitable” judgment. Further, the left hand makes a “positive” judgment, the right hand makes a “negative” judgment.

When you are ready, press the “1” button to start the exercise. The formal experiment will then begin.

**Instructions**

**Testing phase.**

Welcome back to our experiment!

Next, there will be a series of adjectives appearing on the screen, some appeared in the previous evaluation stage, and some are new words. Please do your best to quickly determine whether these words appeared in the previous evaluation stage. If you think the word has appeared in the evaluation stage, press the “f” button. If you do not believe that they appeared in the previous stage, press the “j” button. For words that you think have appeared in the evaluation stage, make a further judgment about whether you “remember” or “know” the word.

“Remember” means that you not only recognize the word as being in the evaluation phase, but that you also recall some of the elements and details that occurred when you saw the word. “Know” means that you think you have seen the word in the evaluation stage, and it feels familiar, but you cannot recall the specific details. Please press the “q” button for “remember” and press the “p” button for “know.”

Note: Please respond to all of the above decisions as soon as possible!

When you are ready, press the “1” button to start the exercise. The formal experiment will then begin.

**Appendix C: Materials of Experiment 3**

**Questionnaire**

**Product Involvement Inventory for Laptops**

Please answer the following 10 questions about **laptops**. Please click “√” on the corresponding option. For you, the **laptop** is:

| Very important | 1 | 2 | 3 | 4 | 5 | 6 | 7 | Very unimportant |
| --- | --- | --- | --- | --- | --- | --- | --- | --- |
| Very boring | 1 | 2 | 3 | 4 | 5 | 6 | 7 | Very interesting |
| Very related to me | 1 | 2 | 3 | 4 | 5 | 6 | 7 | None of my business |
| Very exciting | 1 | 2 | 3 | 4 | 5 | 6 | 7 | Very unexciting |
| It doesn’t make any sense to me | 1 | 2 | 3 | 4 | 5 | 6 | 7 | It means a lot to me |
| Very moving | 1 | 2 | 3 | 4 | 5 | 6 | 7 | Very unmoving |
| Very attractive | 1 | 2 | 3 | 4 | 5 | 6 | 7 | Very normal |
| Very worthless | 1 | 2 | 3 | 4 | 5 | 6 | 7 | Very valuable |
| I’m very involved | 1 | 2 | 3 | 4 | 5 | 6 | 7 | I’m not involved |
| Very unnecessary | 1 | 2 | 3 | 4 | 5 | 6 | 7 | Very necessary |

**Product Involvement Inventory for Shower Gels**

Please answer the following 10 questions about **shower gels**. Please click “√” on the corresponding option. For you, **shower gel** is:

| Very important | 1 | 2 | 3 | 4 | 5 | 6 | 7 | Very unimportant |
| --- | --- | --- | --- | --- | --- | --- | --- | --- |
| Very boring | 1 | 2 | 3 | 4 | 5 | 6 | 7 | Very interesting |
| Very related to me | 1 | 2 | 3 | 4 | 5 | 6 | 7 | None of my business |
| Very exciting | 1 | 2 | 3 | 4 | 5 | 6 | 7 | Very unexciting |
| It doesn’t make any sense to me | 1 | 2 | 3 | 4 | 5 | 6 | 7 | It means a lot to me |
| Very moving | 1 | 2 | 3 | 4 | 5 | 6 | 7 | Very unmoving |
| Very attractive | 1 | 2 | 3 | 4 | 5 | 6 | 7 | Very normal |
| Very worthless | 1 | 2 | 3 | 4 | 5 | 6 | 7 | Very valuable |
| I’m very involved | 1 | 2 | 3 | 4 | 5 | 6 | 7 | I’m not involved |
| Very unnecessary | 1 | 2 | 3 | 4 | 5 | 6 | 7 | Very necessary |

**Questionnaire about laptop brand preference**

Thank you for participating in this survey! All answers to this questionnaire will be kept strictly confidential and the results will be a conclusive report that will not reveal any information about you. The results of this study will only be used for academic research and will not be used for any commercial purposes. This survey is not a test, and there are no “right” or “wrong” answers. The effectiveness of the research results depends primarily on whether you reflect your actual opinion. Therefore, we hope that you can express your opinions very carefully, and answer every question, which is very important for our study.

Gender: _________

Age: ___________

The following are the laptop brands on the market:

**Lenovo, Dell, Hewlett-Packard, Asus, Apple, Raytheon,**

**BenQ, Great Wall, Sony, Toshiba, Haier, Samsung,**

**Tsinghua Tongfang, ThinkPad, MIUI, Digital China, Fujitsu, Panasonic**

Please select your high- and low-preference laptop brands from the above brands, fill in the blank below and complete the following questionnaire.

**My high-preference laptop brand is__________________.**

1. I like this laptop brand very much.

Strongly Disagree 1 2 3 4 5 6 7 Strongly Agree

**My low-preference laptop brand is__________________.**

2. I like this laptop brand very much.

Strongly Disagree 1 2 3 4 5 6 7 Strongly Agree

**Questionnaire about shower gel brand preference**

Thank you for participating in this survey! All answers to this questionnaire will be kept strictly confidential and the results will be a conclusive report that will not reveal any information about you. The results of this study will only be used for academic research and will not be used for any commercial purposes. This survey is not a test, and there are no “right” or “wrong” answers. The effectiveness of the research results depends primarily on whether you reflect your actual opinion. Therefore, we hope that you can express your opinions very carefully, and answer every question, which is very important for our study.

Gender: _________

Age: ___________

The following are the shower gel brands on the market:

**Dove, Kustie, Six God, Safeguard, Johnson & Johnson, Lux, Shiseido,**

**LYNX, Avon, CAMAY, Watsons, Dettol, Sebamed,**

**Lifebuoy, Walch, Olay, Nivea, Nature’s Gate, Adidas, Maxam**

Please select your high- and low-preference shower gel brands from the above brands, fill in the blank below and complete the following questionnaire.

**My high-preference shower gel brand is__________________.**

1. I like this shower gel brand very much.

Strongly Disagree 1 2 3 4 5 6 7 Strongly Agree

**My low-preference shower gel brand is__________________.**

2. I like this shower gel brand very much.

Strongly Disagree 1 2 3 4 5 6 7 Strongly Agree

**Brand Adjectives**

| Table C  *The list of adjectives of brand in experiment 3* | | | |
| --- | --- | --- | --- |
| 1 | 2 | 3 | 4 |
| safe | hygienic | desirable | necessary |
| pure | chaste | flashy | top-level |
| noble | potent | attractive | efficient |
| comfortable | economical | frequently-used | modern |
| delicate | rapid | outstanding | credible |
| relieved | user-friendly | pretty | clean |
| excellent | shining | fashionable | newfangled |
| perfect | best | high-end | retro |
| pleasant | terrific | upmarket | classic |
| practical | healthy | transcendental | easy |
| practical | gorgeous | inexpensive | decent |
| cost-efficient | fresh | approving | distinguished |
| secure | known | cute | saving |
| wholesome | smooth | eligible | environmental |
| substantial | effective | economic | breathtaking |
| profitable | glorious | generous | exquisite |
| smug | shoddy | dark | false |
| arduous | dirty | loathe | wasteful |
| tortious | bad | sick | luxurious |
| dim | wrong | wizened | nauseating |
| unwell | sporty | lackluster | turbid |
| lower | exaggerated | dingy | mean |
| incomplete | costly | fake | absurd |
| old | redundant | hard | worse |
| lapsed | vicious | counterintuitive | sorry |
| wicked | stuffy | clumsy | complicate |
| arrogant | rough | common | susceptible |
| rigid | scornful | fooling | illegal |
| lawless | tacky | damaging | deceptive |
| adulterate | indelicate | useless | weak |
| ugly | low-grade | terrible | dismal |
| degraded | stodgy | malformed | trivial |

**Instructions**

**Learning phase.**

Welcome to our experiment!

This is an adjective evaluation experiment, and you will need to determine whether each word is appropriate for describing a particular object or whether it is a positive or negative word. The experiment starts with a red “+” sign on the computer screen to remind you where to look. Then, you will be presented the specific object of the adjective evaluation, which comes from the questionnaire you filled in before. After that comes the adjective. According to your own perceptions, please quickly determine if the adjective is suitable to describe the particular object by answering “yes” or “no.” If you think the adjective fits the description, press the “f” button for “yes” and press the “j” button for “no” if the adjective does not fit the object. The process of judging the valence of adjectives is similar to this. If you feel the word has a positive meaning, press “f,” if you feel the meaning is negative, press “j.” The adjectives are only presented for a short time, so please pay close attention and make your judgment.

If you understand the above instructions, please sit down. Then put both hands on the keyboard, with the left hand index finger on the “f” key, the right hand index finger on the “j” key, which are the keys required to answer the questions. Remember that the left hand makes the “suitable” judgment, and the right hand makes the “unsuitable” judgment. Further, the left hand makes a “positive” judgment, the right hand makes a “negative” judgment.

When you are ready, press the “1” button to start the exercise. The formal experiment will then begin.

**Testing Phase.**

Welcome back to our experiment!

Next, there will be a series of adjectives appearing on the screen, some appeared in the previous evaluation stage, and some are new words. Please do your best to quickly determine whether these words appeared in the previous evaluation stage. If you think the word has appeared in the evaluation stage, press the “f” button. If you do not believe that they appeared in the previous stage, press the “j” button. For words that you think have appeared in the evaluation stage, make a further judgment about whether you “remember” or “know” the word.

“Remember” means that you not only recognize the word as being in the evaluation phase, but that you also recall some of the elements and details that occurred when you saw the word. “Know” means that you think you have seen the word in the evaluation stage, and it feels familiar, but you cannot recall the specific details. Please press the “q” button for “remember” and press the “p” button for “know.”

Note: Please respond to all of the above decisions as soon as possible!

When you are ready, press the “1” button to start the exercise. The formal experiment will then begin.
